# Interneuron-Driven Ictogenesis in the 4-Aminopyridine Model: Depolarization Block and Potassium Accumulation Initiate Seizure-like Activity

**DOI:** 10.3390/ijms26146812

**Published:** 2025-07-16

**Authors:** Elena Yu. Proskurina, Julia L. Ergina, Aleksey V. Zaitsev

**Affiliations:** Laboratory of Molecular Mechanisms of Neural Interactions, Sechenov Institute of Evolutionary Physiology and Biochemistry of RAS, 194223 Saint Petersburg, Russia; julia.ergina@gmail.com

**Keywords:** epilepsy, seizure initiation, fast-spiking interneurons, 4-AP model, depolarization block, potassium dynamics, entorhinal cortex, hippocampal CA1, in vitro electrophysiology, disinhibition

## Abstract

The mechanisms of ictal discharge initiation remain incompletely understood, particularly the paradoxical role of inhibitory fast-spiking interneurons in seizure generation. Using simultaneous whole-cell recordings of interneurons and pyramidal neurons combined with extracellular [K^+^]_o_ monitoring in mouse entorhinal cortex-hippocampal slices (4-aminopyridine model of epileptiform activity), we identified a critical transition sequence: interneurons displayed high-frequency firing during the preictal phase before entering depolarization block (DB). DB onset coincided with the peak of rate of extracellular [K^+^] accumulation. Pyramidal cells remained largely silent during interneuronal hyperactivity but started firing within 1.1 ± 0.3 s after DB onset, marking the transition to ictal discharges. This consistent sequence (interneuron DB → [K^+^]_o_ rate peak → pyramidal cell firing) was observed in 100% of entorhinal cortex recordings. Importantly, while neurons across all entorhinal cortical layers synchronously fired during the first ictal discharge, hippocampal CA1 neurons showed fundamentally different activity: they generated high-frequency interictal bursts but did not participate in ictal events, indicating region-specific seizure initiation mechanisms. Our results demonstrate that interneuron depolarization block acts as a precise temporal switch for ictogenesis and suggest that the combined effect of disinhibition and K^+^-mediated depolarization triggers synchronous pyramidal neuron recruitment. These findings provide a mechanistic framework for seizure initiation in focal epilepsy, highlighting fast-spiking interneurons dysfunction as a potential therapeutic target.

## 1. Introduction

Epileptic seizure initiation, particularly the transition from interictal to ictal states, remains a fundamental challenge in neuroscience. While impaired inhibition has long been considered central to ictogenesis, accumulating evidence reveals a paradoxical role of GABAergic interneuron hyperactivity in triggering ictal discharges across multiple in vitro models [1,2,3,4] and human tissue preparations [5,6]. This paradox is especially prominent in the 4-aminopyridine (4-AP) model, where synchronized bursts of fast-spiking interneurons coincide with extracellular potassium ([K^+^]_o_) elevations—a potential trigger for ictal transitions [7,8]. These observations challenge conventional views of seizure mechanisms and demand closer examination of how inhibitory networks facilitate ictogenesis.

Recent studies have proposed several non-mutually exclusive hypotheses to explain this phenomenon [9]. The first suggests that synchronized interneuron activity drives substantial potassium efflux, depolarizing pyramidal cells (Pyr) and enabling their synchronous recruitment [10,11,12]. The second hypothesis emphasizes excitatory GABAergic signaling due to chloride accumulation and shifts in GABA reversal potential [13,14,15]. A third framework highlights depolarization block (DB) of interneurons through sodium channel inactivation as a critical network disinhibition mechanism [16,17,18,19]. While optogenetic and pharmacological studies support each mechanism in isolation [4,20], their spatiotemporal interplay during spontaneous seizure initiation remains unresolved. Crucially, the precise cellular dynamics—particularly how these processes interact at the moment of interictal-to-ictal transition—have not been captured with sufficient temporal resolution.

To address these gaps, we employed simultaneous whole-cell recordings of fast-spiking interneurons and pyramidal cells combined with real-time [K^+^]_o_ monitoring in mouse entorhinal cortex (EC)-hippocampal slices. This approach allowed us to reconstruct, with millisecond precision, the cascade of events leading to ictal discharges. We focused on three pivotal questions: (1) the temporal sequence of fast-spiking interneuron depolarization block (DB) relative to ictal onset, (2) the correlation between [K^+^]_o_ dynamics and pyramidal cell activation, and (3) layer- and region-specific differences (deep vs. superficial EC; EC vs. CA1). Our findings reveal a hybrid mechanism where fast-spiking interneuron DB and potassium accumulation jointly gate the transition to ictal states, providing a unified framework for understanding focal seizure initiation.

## 2. Results

### 2.1. Distinct Firing Patterns of Fast-Spiking Interneurons and Pyramidal Cells During Preictal and Ictal Phases in Deep EC

**Figure 1:**
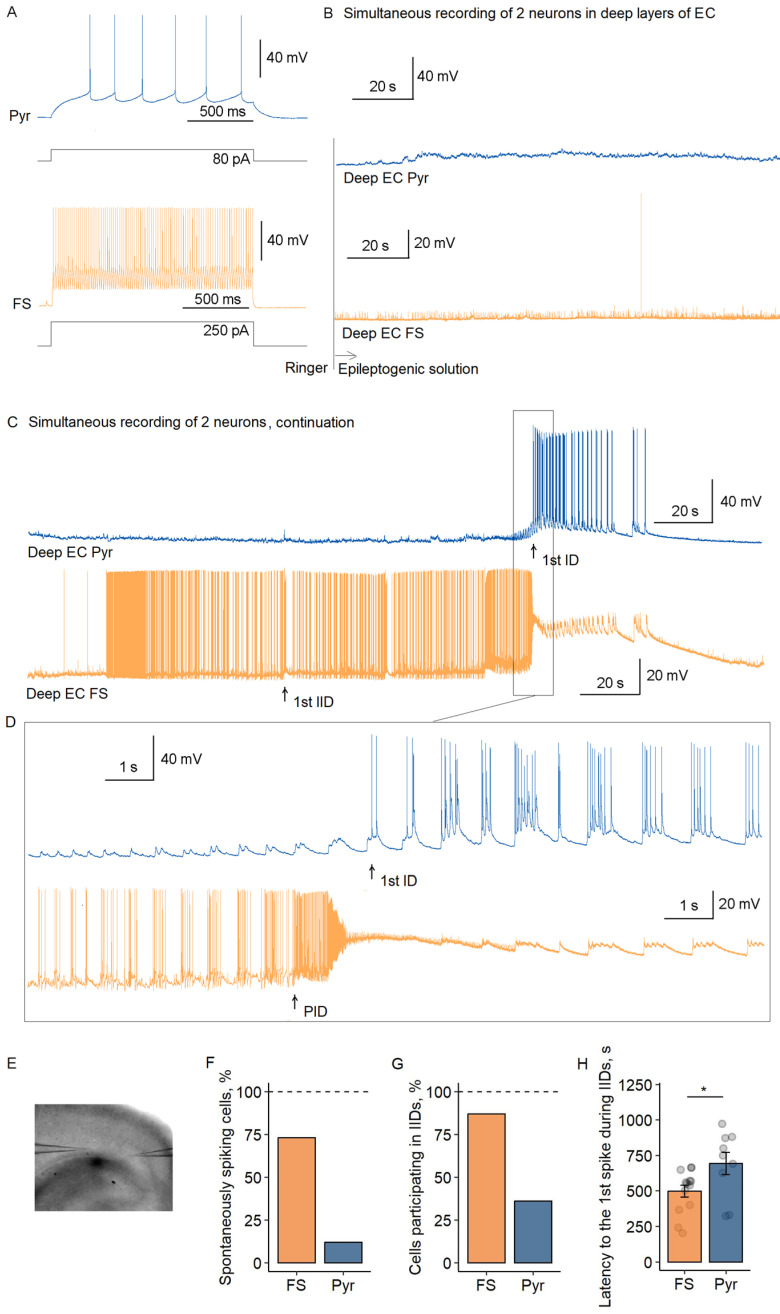
Epileptiform activity generation in deep EC layers following transition from Ringer’s to 4-AP-containing epileptogenic solution. (**A**) Representative firing patterns of a pyramidal cell (Pyr, blue) and fast-spiking interneuron (FS, orange) in response to depolarizing current steps in control conditions. (**B**) Simultaneous membrane potential recordings from a pyramidal cell (top) and fast-spiking interneuron (bottom) during solution transition. (**C**) Continuation of the recording showing first ictal discharge (ID) development. (**D**) High-temporal resolution view of preictal-to-ictal transition. (**E**) Photograph of the brain slice with recording electrode positions marked. (**F**,**G**) Percentage of fast-spiking interneurons (orange) and pyramidal cells (blue) exhibiting (**F**) spontaneous spiking before IIDs and (**G**) participation in interictal discharges (IIDs). (**H**) Latency to the first spike during IIDs (* *p* < 0.05, Student’s *t*-test; circles: individual values; whiskers: SEM).

To investigate ictal discharge initiation in the 4-AP model, we performed simultaneous whole-cell recordings of fast-spiking interneurons and pyramidal cells (*n* = 15 pairs) or pairs of pyramidal cells (*n* = 5) in deep EC layers. Neurons were identified by morphological features (fast-spiking interneurons: round or oval cell body, no apical dendrite; pyramidal cells: pyramidal soma with apical dendrite) and firing patterns (interneurons: narrow action potentials [APD ≤ 0.9 ms], non-adapting high-frequency firing [≥50 Hz]; Pyr: wide action potentials [APD ≥ 1.2 ms], adapting or long-delay spike trains [15–20 Hz], Figure 1A). Under control conditions (Ringer’s solution), both cell types remained quiescent (Figure 1B). Perfusion with epileptogenic solution (containing 100 μM 4-AP) initiated the sporadic firing of most fast-spiking interneurons (11/15) within 430 ± 70 s (Figure 1C–F), which progressed to stereotypic interictal discharges (IIDs) in 87% of cases (13/15; latency: 499 ± 42 s, Figure 1G,H). IIDs occurred at 0.040 ± 0.004 Hz, with burst durations of 0.78 ± 0.14 s (*n* = 13). In contrast, pyramidal cells displayed minimal activity during interictal periods. Only 3/25 and 9/25 pyramidal cells exhibited sparse spiking before and during IIDs, respectively. Pyramidal cells were involved in spike generation during IIDs significantly later than fast-spiking interneurons (interneurons: 499 ± 42 s vs. Pyr: 694 ± 77 s; *p* < 0.05, Student’s *t*-test; Figure 1H). When active during IIDs, pyramidal cell spikes lacked temporal coordination, unlike the synchronized bursts of interneurons.

**Figure A1:**
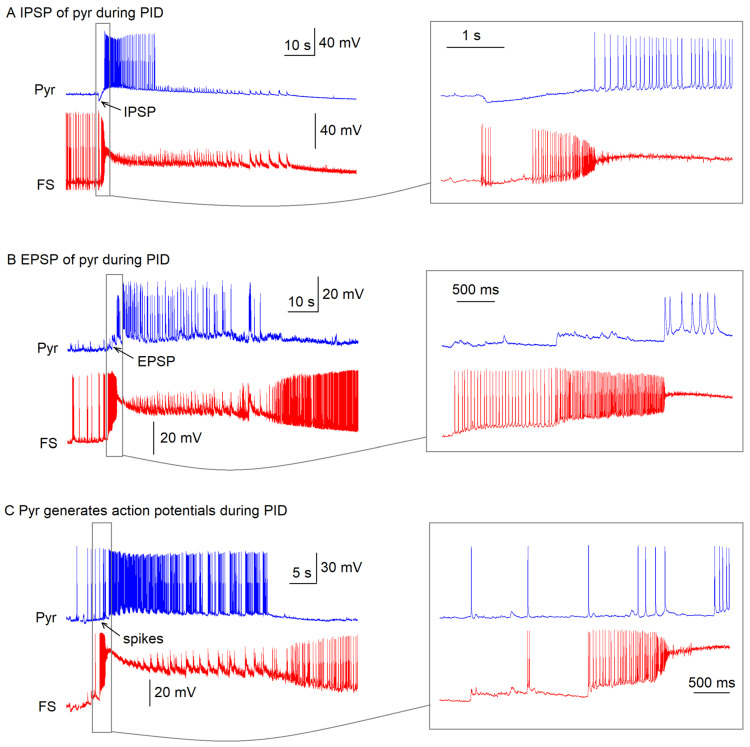
Pyramidal cells exhibited either inhibitory postsynaptic potentials (**A**), excitatory postsynaptic potential (**B**), or sparse action potentials (**C**) during preictal discharge (PID). Representative examples of these 3 cases are presented with simultaneous recordings of the membrane potentials of a pyramidal cell (Pyr, blue curve) and fast-spiking interneuron (FS, red curve). Scaled preictal discharges are presented on the right panels.

Ictal discharges were defined as sustained (>10 s) synchronized events involving both interneurons and pyramidal cells, distinguished from IIDs by duration and network participation. The ictal onset was operationally defined by the first action potential in pyramidal cells following preictal activity (Figure 1C,D). In all paired recordings (*n* = 15), ictal discharges were preceded by a stereotypic sequence: fast-spiking interneurons initially showed high-frequency firing (100% of cells, *n* = 15) before entering a depolarization block state lasting 51 ± 10 s (*n* = 15), while pyramidal cells exhibited either inhibitory postsynaptic potentials (33%, *n* = 5), excitatory postsynaptic potentials (40%, *n* = 6), or sparse action potentials (27%, *n* = 4) during this preictal phase (Appendix A, Figure A1). The interval between preictal and ictal onset was 1.6 ± 0.2 s (*n* = 15 pairs).

During the first ictal discharge, pyramidal cells exhibited sustained firing (duration: 42 ± 7 s; mean frequency of spikes in tonic phase: 18 ± 2 Hz), while fast-spiking interneurons remained in persistent depolarization block. The stereotypic sequence (interneurons DB → pyramidal cell recruitment) across recordings strongly supports disinhibition as the principal mechanism for interictal-to-ictal transition in this model.

### 2.2. Laminar-Specific Dynamics of Ictal Initiation in Entorhinal Cortex

To examine potential laminar differences in ictal generation, we extended our recordings to superficial EC layers (*n* = 9 slices). Simultaneously with pyramidal neurons from deep layers, we recorded fast-spiking interneurons (*n* = 4) or pyramidal cells (*n* = 5) from superficial layers. Cell identity was confirmed through morphological and electrophysiological criteria as described above.

**Figure 2:**
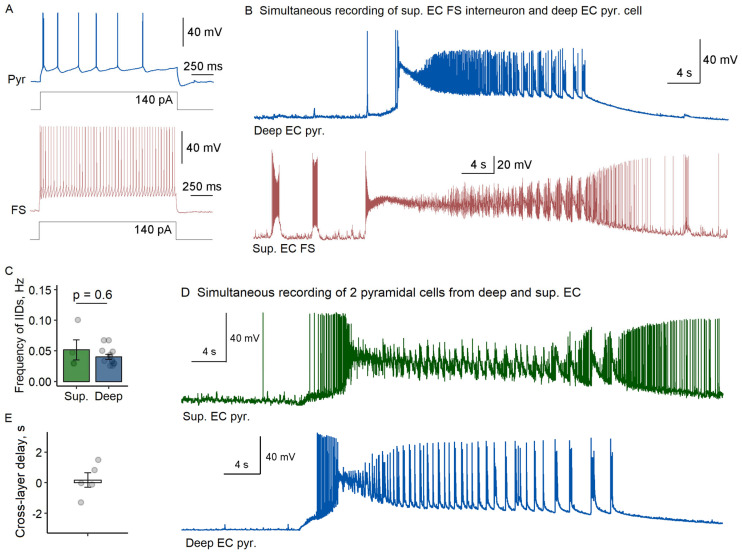
Laminar similarity in ictal discharge initiation between superficial (Sup.) and deep EC layers. (**A**) Representative firing patterns of a deep EC pyramidal cell (Pyr, blue) and sup. EC fast-spiking interneuron (FS, red) in response to depolarizing current steps in control conditions. (**B**) Simultaneous membrane potential recordings from a deep layer pyramidal cell (Pyr, green) and superficial layer fast-spiking interneuron (FS, red). (**C**) Interictal discharge (IID) frequency comparison between layers (*p* = 0.6, Welch’s *t*-test). (**D**) Representative dual recording of pyramidal cells across EC layers during ictal transition. (**E**) Cross-layer synchronization delay. Each circle represents an individual measurement.

Our comparative analysis revealed striking similarities between layers. Fast-spiking interneurons in superficial layers displayed preictal firing patterns nearly identical to their deep layer counterparts, initiating interictal discharges at comparable frequencies (superficial: 0.052 ± 0.017 Hz vs. deep: 0.040 ± 0.004 Hz; *p* = 0.6, Welch’s *t*-test, Figure 2A–C). Most significantly, all recorded fast-spiking interneurons in superficial layers entered depolarization block prior to ictal onset, mirroring that observed in deep layers.

The synchrony between layers became particularly evident during ictal transitions. Cross-layer recordings of pyramidal cells demonstrated coordinated activation, with minimal delay between deep and superficial layers (180 ± 470 ms; Figure 2D,E). This laminar synchrony suggests that the transition from interictal to ictal states propagates from one microcolumn to another in EC rather than spreading sequentially across layers.

### 2.3. Differential Engagement of Hippocampal CA1 During EC-Generated Ictal Activity

**Figure 3:**
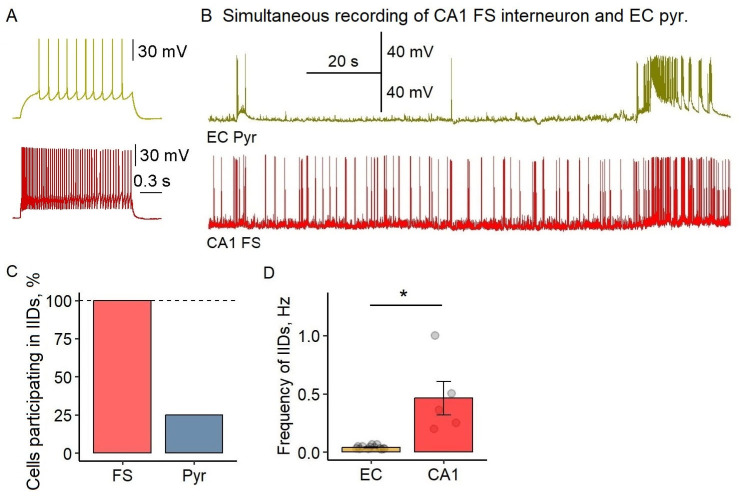
Differential involvement of hippocampal CA1 neurons during EC-generated ictal activity. (**A**) Representative firing patterns of an EC pyramidal cell (top, yellow) and CA1 fast-spiking interneuron (FS, bottom, red) in response to current steps (Ringer’s solution). (**B**) Simultaneous recordings of the EC pyramidal cell (top trace) and CA1 fast-spiking interneuron (bottom trace) showing independent activity patterns. (**C**) Percentage of CA1 fast-spiking interneurons (red) and CA1 pyramidal cells (blue) participating in interictal discharges (IIDs). (**D**) IID frequency comparison between CA1 and EC regions (* *p* < 0.05, Welch’s *t*-test). Each circle represents an individual measurement.

Next, we performed nine cross-regional recordings, pairing CA1 interneurons with EC pyramidal cells (*n* = 5) and CA1 with EC pyramidal cells (*n* = 4). Our recordings revealed striking differences in network engagement between entorhinal cortex and hippocampal CA1 regions during ictogenesis (Figure 3). While fast-spiking interneurons in CA1 exhibited remarkably high interictal discharge frequencies (0.46 ± 0.14 Hz)—an order of magnitude greater than in EC (0.040 ± 0.004 Hz; *p* < 0.05, Welch’s *t*-test)—those bursts occurred independently of EC activity patterns. Only one of the CA1-EC recorded pairs showed any temporal correlation between regions, suggesting functional decoupling during early epileptiform events.

The most notable finding emerged during fully developed ictal discharges in EC, where CA1 pyramidal cells remained strikingly silent despite robust network synchronization in entorhinal cortex. Of the four recorded CA1 pyramidal cells, only one exhibited sparse, unpatterned spiking that lacked the characteristic depolarization seen in EC neurons. This resistance to ictal recruitment contrasted sharply with the high-frequency bursting maintained by CA1 interneurons (*n* = 5), demonstrating region-specific network dynamics in seizure initiation.

These observations reveal that CA1 networks maintain their own distinct pattern of epileptiform activity, which shows minimal correlation with developing ictal discharges in EC during the initial phase of seizure-like activity in our slices. The temporal dissociation between regions is particularly notable, with EC consistently entering ictal states while CA1 preserves its independent interictal bursting pattern. While these findings characterize the initial network dynamics following 4-AP application, we note that prolonged epileptiform activity may alter EC-CA1 interactions, as suggested by reports of late-onset hippocampal involvement in extended recordings [8]. The consistent precedence of EC ictal discharges in our experiments (observed in 100% of slices) highlights the region-specific initiation of seizure-like activity in this model.

### 2.4. Depolarization Block of Fast-Spiking Interneurons Precisely Precedes Ictal Discharge Onset

**Figure 4:**
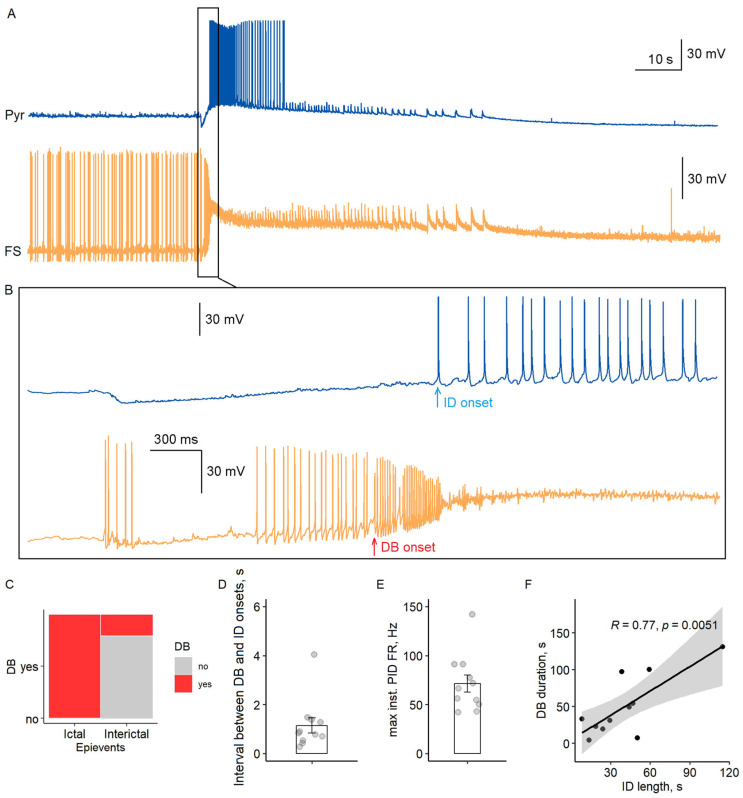
Precise timing of fast-spiking interneuron (FS) depolarization block (DB) relative to ictal discharge onset. (**A**) Simultaneous recording of a pyramidal cell (Pyr, top) and fast-spiking interneuron (bottom) in entorhinal cortex during transition to ictal activity. (**B**) High-temporal resolution view of DB onset (red arrow) and subsequent pyramidal cell activation (blue arrow). (**C**) Depolarization block occurrence during interictal vs. ictal events (*n* = 15 recordings; *p* < 0.001, Fisher’s exact test). (**D**) Interval between DB onset and pyramidal cell first action potential (*n* = 11; mean ± SEM). (**E**) Maximal instantaneous firing rate of fast-spiking interneurons during preictal discharges. (**F**) Correlation between DB duration and ictal discharge length (*r* = 0.77, *p* = 0.005). Each circle represents an individual measurement.

Our paired recordings revealed a consistent temporal sequence in ictal discharge initiation (Figure 4A,B). Fast-spiking interneurons entered a sustained DB state during all observed ictal events (*n* = 15/15 recordings), while DB occurred in only 27% of interictal discharges (3/11; Fisher’s exact test, *p* < 0.001). This striking difference suggests that DB serves as a critical switch for ictal transition.

The transition followed a precise temporal pattern: fast-spiking interneurons first exhibited high-frequency preictal firing (71 ± 9 Hz maximal rate, *n* = 11), then entered DB (membrane potential −33 ± 1 mV, duration 51 ± 10 s), followed by pyramidal cell activation within 1.1 ± 0.3 s (Figure 4C–E). Notably, DB duration strongly correlated with ictal discharge length (Pearson’s *r* = 0.77, *p* < 0.01, Figure 4F), indicating that sustained disinhibition maintains network synchrony during seizures.

### 2.5. Depolarization Block and Potassium Dynamics Converge to Initiate Ictal Transitions

**Figure 5:**
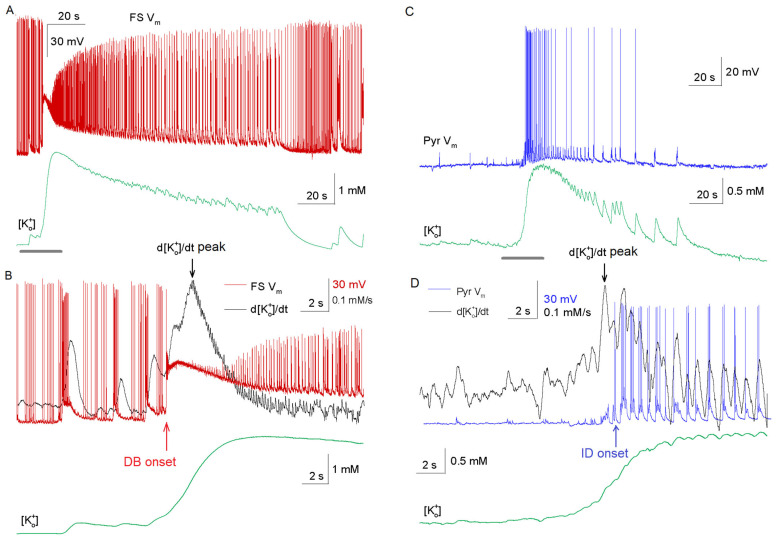
Extracellular potassium ([K^+^]_o_) dynamics during ictal transition. (**A**) Simultaneous recording of fast-spiking interneuron membrane potential (FS, red) and [K^+^]_o_ (green) showing coordinated changes. (**B**) High-temporal resolution view of DB onset (red arrow) and d[K^+^]_o_/dt peak (black arrow). (**C**,**D**) Simultaneous recording of pyramidal cell membrane potential (blue) and [K^+^]_o_ (green) showing coordinated changes (black arrow for d[K^+^]_o_/dt peak, blue arrow for ID onset).

Given the hypothesized role of potassium accumulation in epileptogenesis [11], we measured extracellular potassium concentration ([K^+^]_o_) alongside pyramidal cell activity to determine whether or not [K^+^]_o_ changes correlate with ictal onset (*n* = 10 slices) and alongside FS interneuron activity to study the interdependence of depolarization block and [K^+^]_o_ changes (*n* = 10 slices). Simultaneous recordings in deep EC layers revealed transient [K^+^]_o_ surges during both interictal and ictal discharges (Figure 5A). To standardize analysis, we calculated the rate of [K^+^]_o_ change (d[K^+^]_o_/dt), which peaked before ictal onset in 80% of cases (8/10; Figure 5C,D). Notably, this peak is preceded by FS interneurons DB onset in 100% of slices (*n* = 10, Figure 5B), suggesting a link between interneuron hyperactivity, potassium efflux, and subsequent pyramidal cell activation. Exceptions (2/10) occurred when pyramidal cells fired during pre-ictal discharges. These observations align with the proposed mechanism where interneuron-driven potassium efflux facilitates pyramidal cell excitation [7,11].

## 3. Discussion

Our study provides direct experimental evidence that ictal discharges in the 4-AP model are initiated through a stereotypic sequence of events involving the high-frequency firing of fast-spiking interneurons accompanied by [K^+^]_o_ accumulation, consecutive FS interneuron DB, and, finally, pyramidal cell recruitment. This transition was observed in 100% of EC recordings, with fast-spiking interneurons entering DB 1.1 ± 0.3 s earlier than pyramidal cells start generating APs. The peak rate of [K^+^]_o_ rise occurred when FS interneurons were already in the state of depolarization block, but the pyramidal cells had not yet begun to fire. Notably, this sequence of events was consistent across superficial and deep EC layers, while hippocampal CA1 activity was dissimilar, with fast-spiking interneurons generating high-frequency interictal bursts during ictal events in EC.

### 3.1. Hybrid Mechanism: Disinhibition and Potassium Dynamics Synergize to Initiate Seizures

Our results establish a unified framework for ictogenesis, where fast-spiking interneuron dysfunction acts as a temporal switch. The dual requirement of DB (disinhibition) and [K^+^]_o_-mediated depolarization challenges classical “pure disinhibition” models and highlights the interplay between neural and ionic dynamics.

Our findings align with and extend the well-established hypothesis that synchronized interneuron activity drives ictal discharges through potassium efflux and pyramidal cell depolarization [10,15]. In vitro studies, including 4-AP models, suggest that the high-frequency firing of fast-spiking interneurons elevates [K^+^]_o_ via GABA_A_ receptor activation and subsequent KCC2 cotransporter activity [7,8,21]. This [K^+^]_o_ accumulation depolarizes pyramidal cells, shifting the network toward synchronization—a mechanism corroborated by our observation of peak rate of [K^+^]_o_ rise coinciding with fast-spiking interneuron DB and preceding pyramidal cell recruitment (Figure 5).

However, our data refine this hypothesis by revealing a critical temporal hierarchy: DB of fast-spiking interneurons acts as a gate, ensuring that [K^+^]_o_ accumulation alone is insufficient for ictogenesis without concurrent disinhibition. While prior work emphasizes potassium-driven depolarization [7], we show that most pyramidal cells remain silent during the hyperactivity of fast-spiking interneurons unless FS DB occurs (Figure 4). This suggests that potassium efflux enables synchronization but requires the collapse of inhibitory restraint (via DB) to trigger the transition. Such a hybrid mechanism reconciles conflicting models by integrating ionic and network dynamics.

### 3.2. Chloride Homeostasis and GABAergic Signaling: Revisiting the Excitatory Inhibition Hypothesis

Our results challenge the prevailing view that excitatory GABAergic signaling—mediated through chloride accumulation and shifts in the GABA reversal potential (*E*_GABA_)—serves as the primary driver of ictal discharge initiation. While our whole-cell recordings maintained *E*_GABA_ at −65 mV, this value falls well within the physiological range for mature pyramidal cells [22]. Importantly, these findings are corroborated by extracellular recording studies from Ziburkus et al. (2006) and Trevelyan’s group (2015), which similarly demonstrate pyramidal cell quiescence during preictal interneuron hyperactivity in unperturbed chloride conditions [18,23].

The maintenance of inhibitory GABAergic tone in our experiments aligns with established mechanisms of chloride homeostasis in adult neural networks. Under physiological conditions, KCC2 activity typically maintains *E*_GABA_ between −70 and −75 mV in mature pyramidal neurons [22], ensuring GABAergic inhibition predominates. While prior work demonstrates that intense interneuron activity can elevate intracellular chloride concentration ([Cl^-^]_i_) and depolarize *E*_GABA_ [13,23], we observed only few pyramidal cells spiking during preictal fast-spiking interneuron hyperactivity (Figure 4), arguing against early GABA-mediated excitation. Instead, most pyramidal cells remained silent until fast-spiking interneurons entered DB, suggesting that disinhibition, rather than excitatory GABA, gates the transition.

Our conclusions are further reinforced by previously reported results examining the effects of KCC2, the principal chloride extruder responsible for maintaining inhibitory GABAergic tone. In mature neural networks, pharmacological impairment of KCC2 function (e.g., via furosemide administration) unexpectedly suppresses ictal activity [21]. This paradoxical effect strongly suggests that chloride accumulation by itself is insufficient to drive seizure initiation in adult tissue—a finding that directly parallels our observation of pyramidal cell quiescence during preictal interneuron hyperactivity. The clinical relevance of this mechanism is underscored by human tissue studies demonstrating that KCC2 downregulation correlates with depolarizing GABA responses only during established ictal phases [24]. While our data clearly indicate that disinhibition gates the initial transition to seizure activity, we acknowledge the potential synergistic effects between chloride dynamics and [K^+^]_o_ accumulation in sustaining later ictal phases [12].

### 3.3. Depolarization Block of Fast-Spiking Interneurons as a Critical Gate for Ictal Transition

Our results provide compelling support for the DB hypothesis, demonstrating that sodium channel inactivation in fast-spiking interneurons serves as the critical disinhibitory switch for ictal transition. The stereotypic sequence we observed—where fast-spiking interneuron DB consistently preceded both [K^+^]_o_ peaks and pyramidal cell recruitment—aligns with computational models showing that sustained depolarization inactivates voltage-gated sodium channels, silencing interneurons and releasing pyramidal networks from inhibition [16]. This mechanism explains the paradoxical observation that interneuron hyperactivity ultimately promotes seizure initiation: their initial high-frequency firing drives potassium efflux, while subsequent DB removes inhibitory restraint, creating a permissive window for synchronous pyramidal discharge. Notably, the strong correlation between DB duration and ictal discharge length (r = 0.77) suggests that DB maintenance is crucial for seizure sustenance, not just initiation.

The propensity of fast-spiking interneurons to enter DB in the 4-AP model stems from their unique electrophysiological properties and the pharmacological effects of 4-AP on ion channels. Fast-spiking interneurons rely heavily on Kv3-type potassium channels, which enable rapid repolarization and sustain high-frequency firing [25,26], in addition to contributions from other A-type currents mediated by Kv1 and Kv4 subunits [27,28]. When 4-AP blocks these channels, action potentials broaden, and the fAHP diminishes, impairing the neuron’s ability to repolarize efficiently [27]. This prolonged depolarization leads to the cumulative inactivation of voltage-gated sodium (NaV) channels, rendering interneurons incapable of generating further action potentials despite continued excitatory input [29]. As mentioned above, the intense firing of fast-spiking interneurons during preictal phases exacerbates [K^+^]_o_ accumulation, further depolarizing the membrane and accelerating NaV channel inactivation [7,30]. The resulting DB is thus a consequence of disrupted repolarization, sustained depolarization, and compromised NaV channel availability. In contrast to fast-spiking interneurons, pyramidal neurons exhibit relative resistance to DB due to their distinct ion channel composition and lower firing rates [31]. Pyramidal neurons express a diverse array of potassium channels, including Kv1, Kv2, Kv4, and Kv7, some of which are less sensitive to 4-AP, allowing for more effective repolarization, even under hyperexcitable conditions [32,33].

The differential susceptibility of fast-spiking interneurons and pyramidal neurons to DB has profound implications for seizure dynamics. The collapse of inhibitory control due to fast-spiking interneuron DB removes a critical brake on network excitation, facilitating the synchronous recruitment of pyramidal neurons and the transition to ictal states [34].

### 3.4. Region-Specific Mechanisms: Entorhinal Cortex vs. Hippocampal CA1

Our findings highlight a striking divergence in seizure susceptibility between the EC and hippocampal CA1. While EC pyramidal neurons synchronously generate ictal discharges following interneuron DB, CA1 networks remain resistant to ictal recruitment, despite exhibiting high-frequency interictal bursts. This dissociation aligns with prior work showing that ictal events in the 4-AP model preferentially originate in the EC, whereas CA1 activity is often limited to interictal spikes unless driven by upstream regions like CA3 [7,8]. The EC’s propensity for ictogenesis likely stems from its recurrent excitatory connectivity that promotes rapid synchronization. In contrast, CA1’s reliance on Schaffer collateral inputs and strong feed-forward inhibition may act as a filter, preventing local ictal emergence [35,36].

### 3.5. Therapeutic Implications and Unresolved Questions

Our findings suggest several promising therapeutic strategies that are already under investigation. The first strategy involves modulating interneuron activity to prevent their transition into DB. This could be achieved by targeting voltage-gated potassium channels (e.g., Kv3 modulators) stabilizing high-frequency firing in fast-spiking interneurons by promoting rapid repolarization, thereby delaying DB onset [16]. Optogenetic approaches, such as low-frequency co-activation of both excitatory and inhibitory neurons, have also shown efficacy in suppressing ictal discharges while avoiding excessive interneuron depolarization [37]. Closed-loop systems could dynamically adjust stimulation parameters in real-time to counteract DB triggers, such as [K^+^]_o_ surges [12].

The regulation of [K^+^]_o_ dynamics presents another promising therapeutic avenue for controlling seizure activity [38]. Experimental evidence suggests that enhancing astrocytic potassium buffering through Kir4.1 channels could mitigate epileptiform discharges, as these channels are critical for [K^+^]_o_ regulation [39]. For instance, gene therapy approaches overexpressing Kir4.1 in astrocytes have shown potential in restoring potassium homeostasis and reducing neuronal hyperexcitability in models of Huntington’s disease, highlighting its applicability for epilepsy [40]. Pharmacologically, although specific Kir4.1 activators remain elusive, antiepileptic drugs like valproate and phenobarbital indirectly upregulate Kir4.1 expression, suggesting a secondary mechanism of action [38].

While our findings provide mechanistic insights into ictal initiation, several limitations should be acknowledged. First, the use of acute brain slices excludes the influence of long-range connectivity and neuromodulatory systems present in vivo, potentially simplifying network dynamics. Second, the 4-AP model, while widely used, induces a uniform pharmacological disinhibition that may not fully replicate the pathological heterogeneity of human epilepsy. Additionally, our recordings focused primarily on fast-spiking interneurons and pyramidal cells; contributions from other interneuron subtypes or glial mechanisms in potassium buffering remain to be explored. Finally, the translational relevance of these findings requires validation in chronic epilepsy models or human tissue, where structural remodeling and compensatory plasticity may alter the observed dynamics.

## 4. Materials and Methods

### 4.1. Animals

All experimental procedures were approved by the Bioethics Committee of the Sechenov Institute of Evolutionary Physiology and Biochemistry (Protocol No. 1-15/2023, 26 January 2023) and complied with the European Community Council Directive 1986 (86/609/EEC). Adult male C57BL/6 mice aged 3–9 months were housed under controlled conditions (12 h light/dark cycle, ambient temperature, and humidity) with ad libitum access to food and water.

### 4.2. Brain Slice Preparation

Horizontal brain slices (300 µm thickness) containing the entorhinal cortex and hippocampus were prepared as previously described [37], with modifications. Briefly, adult C57BL/6 mice were anesthetized with isoflurane and decapitated, followed by rapid brain extraction in ice-cold cutting solution containing (in mM): 110 N-methyl-D-glucamine (NMDG), 2.5 KCl, 1.2 NaH_2_PO_4_, 10 MgSO_4_, 0.5 CaCl_2_, 25 NaHCO_3_, and 25 D-glucose, continuously oxygenated with 95% O_2_/5% CO_2_. Slices were incubated for ≥1 h at room temperature in standard Ringer solution (in mM: 126 NaCl, 2.5 KCl, 1.25 NaH_2_PO_4_, 1 MgSO_4_, 2 CaCl_2_, 24 NaHCO_3_, 13.32 D-glucose) before electrophysiological recordings.

### 4.3. In Vitro Seizure Model

Epileptiform activity was induced by perfusing brain slices with a pro-epileptic solution containing 100 μM 4-AP in modified Ringer’s solution (composition in mM: 125 NaCl, 3.5 KCl, 1.25 NaH_2_PO_4_, 0.25 MgSO_4_, 2 CaCl_2_, 24 NaHCO_3_, and 13.32 D-glucose). The solution was continuously oxygenated with 95% O_2_/5% CO_2_ and maintained at 30 °C during recordings.

### 4.4. The Whole-Cell Patch-Clamp Recordings

Electrophysiological recordings were performed at 30 °C using either a HEKA EPC-10 amplifier (HEKA Elektronik, Lambrecht, Germany) or a Multiclamp 700B amplifier (Molecular Devices, San Jose, CA, USA). Patch pipettes with resistances of 3–6 MΩ were pulled from borosilicate glass capillaries (World Precision Instruments, Sarasota, FL, USA) using a P-1000 Micropipette Puller (Sutter Instrument, Novato, CA, USA) and filled with intracellular solution containing (in mM) 135 K-gluconate, 10 NaCl, 5 EGTA, 10 HEPES, 4 ATP-Mg, and 0.3 GTP, with pH adjusted to 7.25 using KOH. Neurons were visualized under infrared differential interference contrast optics using a Nikon Eclipse FN1 microscope (Nikon, Tokyo, Japan) equipped with a Grasshopper3 video camera (FLIR Systems, Wilsonville, OR, USA).

Fast-spiking interneurons were identified by their characteristic morphological and electrophysiological properties. Morphologically, these cells exhibited oval or round somata with subtle, poorly distinguishable dendrites when viewed under differential interference contrast. Electrophysiologically, they displayed narrow action potentials with duration at half-amplitude (APD) ≤ 0.9 ms and afterhyperpolarization amplitudes ≥ 15 mV, and could sustain high-frequency firing ≥ 50 Hz in response to depolarizing current steps. Pyramidal cells were distinguished by their pyramidal-shaped somata with prominent apical dendrites, wider action potentials (APD ≥ 1.2 ms), and regular adapting firing patterns or long-delay spike trains in response to depolarization [41,42].

Series resistance was continuously monitored in voltage-clamp mode by analyzing capacitive transients induced by 10 mV hyperpolarizing steps and was maintained below 20 MΩ throughout recordings, with changes limited to ≤30% of initial values. Data were acquired at sampling rates of 30–33 kHz using either PatchMaster v2 software (HEKA Elektronik, Lambrecht, Germany) with an NI USB-6343 A/D converter (National Instruments, Austin, TX, USA) or WinWCP v5 software (University of Strathclyde, Glasgow, UK), with signals filtered at 10 kHz. The membrane potential was corrected offline for liquid junction potential when appropriate.

### 4.5. Analysis of Neuronal Epileptiform Activity

Neuronal activity was analyzed using custom software developed in the Delphi environment. Action potentials were detected based on the membrane potential threshold, defined as the point where the interpolated rate of voltage increase (dV/dt) exceeded 10 V/ms. Depolarization block (DB) was identified when neurons exhibited sustained depolarization (membrane potential > −40 mV) accompanied by a reduction in action potential amplitude below 30 mV, indicating complete cessation of spiking activity.

IIDs were characterized as brief synchronized events lasting less than 1 s, featuring high-frequency firing (≥50 Hz) in fast-spiking interneurons and pronounced depolarization of the interspike membrane potential. These events were consistently associated with transient elevations in extracellular potassium concentration ([K^+^]_o_). The onset of each IID was determined by the first action potential threshold crossing during the event.

For ID, we established three key diagnostic criteria: sustained duration exceeding 10 s, synchronous participation of both fast-spiking interneurons and pyramidal cells, and significant [K^+^]_o_ increases surpassing 3 mM. These events typically progressed through distinct tonic and clonic phases. The precise onset of ictal activity was operationally defined as the first action potential generated by pyramidal cells following preictal activity, providing a consistent marker for temporal analysis.

Our quantitative assessment focused on several critical temporal parameters. These included the latency between fast-spiking interneuron depolarization block onset and subsequent pyramidal cell firing, the duration of depolarization block episodes, the interval separating interictal and ictal events, and the rate of extracellular potassium concentration change (d[K^+^]/dt) during transitional periods. All automatically detected events underwent rigorous visual verification to ensure accuracy. Consistent with our quality control standards, only data from recordings demonstrating stable series resistance (variation < 30% from initial values) were included in the final analysis.

### 4.6. Measurements of the Extracellular Potassium Concentration

Extracellular potassium concentration ([K^+^]_o_) was monitored using potassium-selective microelectrodes fabricated as previously described [43]. Borosilicate glass pipettes were silanized by exposure to hexamethyldisilazane vapor (Sigma-Aldrich, St. Louis, MO, USA) at 220 °C for 90 min. The electrodes were backfilled with 100 mM KCl solution, and their tips were filled with potassium ionophore I cocktail A (Sigma-Aldrich, cat. no. 99311) using gentle suction.

Potassium-sensitive electrode potentials were recorded in current-clamp mode using a Multiclamp 700B amplifier (Molecular Devices). The [K^+^]_o_ was calculated from the electrode voltage V(t) using the Equation (1):

(1) $${\left[K^{+}\right]}_{o}\left(t\right)=2.5\;e^{S*V\left(t\right)}$$

where *S* is the scaling factor, estimated by applying solutions with different [K^+^]_o_ at the tips of ionophore-filled electrodes using a fast application system (HSSE-2/3, ALA Scientific Instruments Inc., Farmingdale, NY, USA). For all electrodes tested, the scaling factor fluctuated within a small range (0.043–0.045), so the average value of 0.044 mV^−1^ was set as the *S* value.

The rate of change of the potassium ions concentration was calculated as d[K^+^]_o_/dt after the RC-filtering of the [K^+^]_o_ signal.

### 4.7. Statistical Analysis

Statistical analyses were performed using RStudio 2024.04.2 (RStudio PBC, Boston, MA, USA). Normality of data distribution was assessed using the Shapiro–Wilk test. For comparisons between two groups with normally distributed data and equal variances, Student’s *t*-test was applied. When variances were unequal (as determined by F-test), Welch’s *t*-test was used instead. Non-parametric comparisons of multiple groups were conducted using the Kruskal–Wallis test followed by Dunn’s post hoc test with Bonferroni correction.

Data are presented as mean ± standard error of the mean (SEM) throughout the manuscript, where “*n*” represents the number of biologically independent samples (brain slices). Correlation analyses were performed using Pearson’s correlation coefficient for normally distributed data. The threshold for statistical significance was set at *p* < 0.05 for all tests.

## Data Availability

The data presented in this study are available on request from the corresponding author.

## Abbreviations

The following abbreviations are used in this manuscript:

4-AP	4-aminopyridine
APD50	action potential duration at half-amplitude
AHP	afterhyperpolarization
CSF	cerebrospinal fluid
DB	depolarization block
EC	entorhinal cortex
FS	fast-spiking interneuron
GABA	γ-aminobutyric acid
IID	interictal discharge
[K^+^]_o_	extracellular potassium concentration
NMDG	N-methyl-D-glucamine
Pyr	pyramidal cell
SEM	standard error of the mean
